# Supplementary material for: Rv1453 is associated with clofazimine resistance in Mycobacterium tuberculosis
Source: Microbiol Spectr. 2023 Aug 24;11(5):e00002-23. doi: 10.1128/spectrum.00002-23 (PMC10580819; doi:10.1128/spectrum.00002-23)
Supplement: Tables S1 to S2 — Details of MTB strains. [file spectrum.00002-23-s0001.docx]

**Supplementary data**

**Table S1. Details of CFZ-susceptible MDR-TB strains.**

| ID | Date of isolation | SRA | Lineage | Medication administration record | Drug resistance profile | CFZ treatment |
| --- | --- | --- | --- | --- | --- | --- |
| 10416 | 201206 | SRR22873610 | 2.2.1 | / | INH RIF SM EMB AM PAS OFX LVFX | No |
| 11264 | 201211 | SRR22873601 | 2.2.1 | INH RIF RFB EMB PZA PA AM CLR CAP LVFX MFX PTO PAS AMX/CLV | INH RIF SM EMB AM PAS OFX LVFX | Yes |
| 12897 | 201308 | SRR22873600 | 2.2.2 | INH RIF EMB | INH SM EMB RIF | No |
| 13385 | 201401 | SRR22873599 | 2.2.2 | / | INH RIF AM PAS OFX LVFX | No |
| 13529 | 201401 | SRR22873598 | 2.2.1.1 | MFX | INH RIF SM OFX LVFX | No |
| 13946 | 201401 | NA | NA | PA INH PTO EMB LVFX AM PAS PZA | INH RIF EMB RFP RFB PAS OFX LVFX | No |
| 13985 | 201402 | NA | NA | INH RIF EMB PZA AM RFP LVFX | INH SM EMB RIF OFX LVFX | No |
| 14092 | 201402 | NA | NA | INH RIF RFP EMB PZA AM LVFX RFB CAP PAS PTO GFX | INH RIF | No |
| 14158 | 201402 | NA | NA | INH RIF RFP EMB PZA SM AM LVFX RFB CAP PTO PA MFX CLR | INH SM EMB RIF RFB AM PAS OFX LVFX RFP | No |
| 14282 | 201403 | NA | NA | INH RIF EMB PZA CAP PAS LVFX PTO AMX/CLV CS | INH SM EMB RIF RFB OFX LVFX RFP | No |
| 14291 | 201403 | NA | NA | INH RIF RFP EMB PZA PA AM LVFX PAS PTO RFB | OFX LVFX INH RIF | Yes |
| 14441 | 201404 | NA | NA | INH RIF LVFX EMB PZA SM AM MFX CAP PAS PTO RFB CS PA | INH EMB RIF RFB AM CAP OFX LVFX RFP | Yes |
| 14550 | 201404 | NA | NA | INH RIF PA EMB PZA PTO LVFX PAS MFX AMX/CLV | RIF INH SM EMB RFB AM OFX LVFX RFP | Yes |
| 14619 | 201404 | NA | NA | INH RIF EMB PZA AM LVFX MFX PA AMX/CLV | INH SM RIF RFB OFX LVFX RFP | No |
| 14822 | 201406 | NA | NA | INH RIF RFB EMB PZA MFX CAP PA PTO CLR | OFX LVFX AM RIF INH EMB RFB CAP RFP SM | No |
| 14862 | 201406 | NA | NA | INH RIF PZA AMX/CLV EMB | OFX LVFX RIF INH EMB RFP SM | No |
| 14959 | 201407 | NA | NA | INH RFP EMB PZA PA KM LVFX | OFX LVFX RIF INH EMB RFB RFP SM | No |
| 15007 | 201407 | NA | NA | INH EMB RIF RFP PZA RFB AM LVFX MFX CLR | INH SM EMB RIF RFB PAS OFX LVFX RFP | Yes |
| 15055 | 201408 | NA | NA | INH RIF RFP RFB EMB PA PZA LVFX AM MFX PTO CLR | INH EMB SM RIF AM OFX RFP | No |
| 15182 | 201409 | NA | NA | INH RIF EMB PZA PA MFX LZD PTO | INH RIF OFX LVFX | Yes |
| 15183 | 201409 | NA | NA | INH RIF PZA AM LVFX RFB PTO CLR | INH EMB RIF RFB OFX LVFX RFP | No |
| 15216 | 201409 | NA | NA | INH RIF EMB PZA PA AM LVFX PAS PTO | OFX LVFX RIF INH EMB RFP SM | No |
| 15285 | 201409 | NA | NA | INH RIF EMB PZA PA AM LVFX RFB PAS PTO CLR | INH SM EMB RIF RFB RFP | No |
| 15321 | 201409 | NA | NA | INH RIF RFP EMB PZA PA MFX | INH SM EMB RIF RFB AM OFX LVFX RFP | No |
| 15344 | 201409 | NA | NA | INH RIF EMB PZA PA LVFX | INH RIF RFB PAS OFX LVFX RFP | No |
| 15346 | 201409 | NA | NA | INH RIF EMB PZA LVFX CAP PAS MFX CS | INH RIF RFB RFP | No |
| 15403 | 201410 | NA | NA | INH RIF EMB PZA AMX/CLV PAS PTO CLR | INH SM EMB RIF RFB OFX LVFX RFP | Yes |
| 15424 | 201410 | NA | NA | INH RIF RFP EMB PZA SM AM LVFX MFX PA PAS CLR CS | OFX LVFX AM RIF INH EMB RFB CAP RFP SM | No |
| 15433 | 201410 | NA | NA | INH RIF EMB PZA CS AM LVFX MFX CAP PAS PTO CLR | OFX LVFX AM RIF INH EMB RFB CAP PAS RFP SM | Yes |
| 15621 | 201410 | NA | NA | INH RIF RFP EMB PZA | RIF INH EMB RFB RFP SM | No |
| 15622 | 201410 | NA | NA | INH RIF EMB PZA LVFX PAS PTO | INH RIF OFX LVFX EMB RFB RFP SM | No |
| 15657 | 201410 | NA | NA | INH RIF EMB PZA | OFX LVFX RIF INH RFB RFP | No |
| 15671 | 201411 | NA | NA | INH RIF RFT EMB PZA SM | OFX LVFX INH RIF PAS | No |
| 15689 | 201411 | NA | NA | INH RIF EMB PZA PTO CS MFX CLR | OFX LVFX RIF INH RFB RFP SM | No |
| 15820 | 201412 | NA | NA | INH RIF EMB PZA | OFX LVFX AM RIF INH RFB CAP RFP SM | No |
| 15833 | 201412 | NA | NA | RIF PZA PAS CLR | RIF INH RFB RFP | No |
| 15914 | 201412 | NA | NA | INH RIF RFP EMB PZA SM AM LVFX MFX PA PAS PTO CLR CS AMX/CLV | INH SM EMB RIF RFB RFP AM OFX LVFX | Yes |
| 15953 | 201412 | NA | NA | INH RIF EMB PZA SM CLR | LVFX OFX RIF INH EMB RFB RFP CAP SM | No |
| 16030 | 201501 | NA | NA | INH RIF EMB PZA | RIF INH RFB RFP | No |
| 16044 | 201501 | NA | NA | INH RIF EMB PZA AM LVFX PAS PTO | OFX LVFX RIF INH EMB PAS | No |
| 16100 | 201501 | NA | NA | INH RIF RFP EMB PZA PA AM LVFX MFX PAS PTO CLR | LVFX MFX RIF INH RFB RFP | Yes |
| 16121 | 201501 | NA | NA | INH RFP EMB PZA PA AM LVFX MFX CAP PAS PTO | RIF INH EMB RFB RFP | Yes |
| 16173 | 201501 | NA | NA | INH RIF EMB PZA RFP LVFX MFX CAP PAS PTO CLR CS RFB AMX/CLV | INH PAS OFX LVFX RFP RIF SM | No |
| 16226 | 201502 | NA | NA | INH RIF EMB SM PA LVFX PTO PAS | INH SM EMB RIF RFB PAS OFX LVFX RFP | No |
| 16381 | 201503 | NA | NA | INH RIF RFP EMB PZA SM AM LVFX MFX PA PAS PTO CLR CS AMX/CLV | OFX LVFX AM RIF INH RFB RFP | Yes |
| 16622 | 201504 | NA | NA | INH RIF EMB PZA PA AM LVFX MFX PTO CLR | RIF INH | No |
| 16761 | 201504 | NA | NA | INH RFP EMB PZA CAP LVFX MFX PAS PTO | INH EMB RIF RFB OFX LVFX RFP | No |
| 16796 | 201504 | NA | NA | / | INH RIF RFP SM | No |
| 16825 | 201505 | NA | NA | EMB PZA AM CLR PAS | AM RIF INH RFB RFP | No |
| 16833 | 201505 | NA | NA | / | INH RIF AM OFX LVFX | No |
| 16846 | 201505 | NA | NA | INH EMB AM LVFX PTO | INH RIF | No |
| 16914 | 201505 | NA | NA | INH RIF EMB PZA PAS AM PZA LVFX | OFX LVFX RIF INH RFB RFP | No |
| 16983 | 201505 | NA | NA | INH RIF EMB PZA AM MFX | INH RIF AM OFX LVFX RFB | No |
| 16995 | 201505 | NA | NA | INH RIF EMB PZA LVFX MFX CAP PAS PTO CS | INH RIF OFX LVFX RFB | No |
| 17080 | 201505 | SRR22873597 | 2.2.1 | INH RIF EMB PZA CS LVFX MFX AMX/CLV PAS PTO CLR | INH RIF SM OFX LVFX RFB | No |
| 17125 | 201506 | NA | NA | INH RIF EMB PZA LVFX | INH SM EMB RIF AM PAS OFX LVFX RFB | No |
| 17139 | 201506 | NA | NA | INH RIF EMB PZA CS AM LVFX MFX CAP PAS PTO CLR | INH SM RIF AM PAS OFX LVFX RFB | No |
| 17141 | 201506 | NA | NA | INH RIF EMB PZA PA CAP PTO MFX GFX CS AM | INH SM RIF RFB OFX RFP | Yes |
| 17143 | 201506 | NA | NA | INH RIF EMB PZA | INH RIF | Yes |
| 17185 | 201506 | NA | NA | INH RIF EMB PZA | INH SM RIF RFP | No |
| 17240 | 201506 | NA | NA | INH RIF EMB PZA LVFX | OFX RIF INH RFB RFP | No |
| 17251 | 201507 | NA | NA | / | INH SM RIF OFX LVFX RFB | No |
| 17305 | 201506 | NA | NA | INH EMB PZA AM LVFX MFX CAP PAS PTO | INH SM RIF RFB AM OFX LVFX RFP | No |
| 17466 | 201507 | NA | NA | INH RIF RFP EMB PZA PA LVFX MFX PAS PTO CLR | INH SM EMB RIF RFB OFX LVFX RFP | No |
| 17548 | 201507 | NA | NA | INH RIF EMB PZA SM AM CLR MFX PTO | INH EMB RIF RFB PAS OFX LVFX RFP | No |
| 17560 | 201507 | NA | NA | INH RIF EMB PZA CAP PAS CLR PA RFP LVFX PTO PZA | INH SM EMB AM OFX LVFX RIF | Yes |
| 17615 | 201507 | NA | NA | INH RIF EMB PZA AM LVFX | INH SM RIF RFB AM OFX LVFX RFP | No |
| 17676 | 201508 | NA | NA | INH RIF EMB PTO | AM INH RIF | No |
| 17696 | 201508 | NA | NA | INH RIF RFP EMB PZA SM AM LVFX MFX CAP PAS PTO CLR PA | INH SM RIF RFB AM PAS OFX LVFX RFP | No |
| 17712 | 201508 | NA | NA | RIF EMB PA PTO LVFX AM RFB | INH EMB RIF AM RFP | No |
| 17801 | 201508 | NA | NA | INH RIF RFP EMB PZA PA AM LVFX MFX PAS PTO | OFX LVFX AM RIF INH EMB RFB PAS RFP SM | No |
| 17815 | 201509 | NA | NA | INH RIF RFP EMB PZA AM MFX PAS | INH SM RIF AM OFX LVFX RFP | No |
| 17854 | 201509 | NA | NA | INH RIF RFP EMB PZA PA AM LVFX MFX CAP PAS PTO CLR AMX/CLV CS LZD | INH SM EMB RIF RFB AM CAP PAS OFX LVFX RFP | Yes |
| 17886 | 201509 | NA | NA | INH RIF CLR EMB PZA AM LVFX MFX | INH SM RIF RFB AM CAP OFX LVFX RFP | No |
| 18022 | 201509 | NA | NA | RIF RFP EMB PZA AM LVFX MFX PAS PA PTO | INH SM RIF RFB AM PAS OFX LVFX RFP | No |
| 18129 | 201509 | NA | NA | INH RIF EMB PZA PA MFX CAP PAS PTO | INH SM EMB RIF AM CAP OFX LVFX RFP | No |
| 18300 | 201510 | NA | NA | INH RIF RFP EMB PZA AM MFX PAS PTO RFB | INH SM RIF PAS OFX LVFX RFP | No |
| 18312 | 201510 | NA | NA | LVFX | INH RIF RFB OFX LVFX RFP | No |
| 18368 | 201510 | NA | NA | INH RIF EMB PZA LVFX MFX PAS PTO | INH RIF RFB OFX LVFX RFP | No |
| 19288 | 201601 | NA | NA | INH RIF EMB PZA | INH SM EMB RIF PAS OFX LVFX RFP RFB | No |
| 19341 | 201602 | NA | NA | INH RIF CS EMB PZA SM AM LVFX PAS PTO LZD | INH SM EMB RIF AM CAP PAS RFP RFB | Yes |
| 19348 | 201602 | NA | NA | INH RIF PZA EMB PA PTO LVFX AM RFP PAS MFX CLR | INH SM RIF AM CAP PAS OFX LVFX RFP RFB | No |
| 19361 | 201602 | NA | NA | INH RIF EMB PZA | INH SM EMB RIF AM OFX LVFX RFB | No |
| 19746 | 201603 | NA | NA | INH RIF EMB PZA PA AM MFX PAS PTO | INH RIF RFP RFB AMX/CLV | Yes |
| 19777 | 201603 | NA | NA | INH RIF EMB PZA AM LVFX PAS | SM INH RIF RFP MFX RFB AMX/CLV | No |
| 19849 | 201604 | NA | NA | INH RIF RFB EMB PZA AM LVFX MFX CAP PAS PTO | INH SM RIF AM OFX LVFX RFP | No |
| 19864 | 201604 | NA | NA | INH RIF EMB PZA LZD MFX CAP PAS PTO | SM INH RIF EMB RFP LVFX AM PTO PA MFX RFB AMX/CLV | Yes |
| 19943 | 201604 | NA | NA | INH RIF EMB PZA AM LVFX MFX LZD PAS PTO CS | SM RIF RFP MFX AMX/CLV LVFX RFB | Yes |
| 20801 | 201607 | NA | NA | INH RIF RFP EMB RFB PZA AMX/CLV MFX PA PTO CLR | INH SM RIF RFP LVFX MFX RFB AMX/CLV | No |
| 20815 | 201607 | NA | NA | INH RIF EMB PZA AM LVFX AMX/CLV CAP PAS PTO CS | SM RIF RFP AM PTO AMX/CLV PAS INH RFB | No |
| 20890 | 201608 | NA | NA | INH RIF EMB PZA LZD LVFX MFX PAS PTO CLR | INH RIF EMB PAS MFX CLR AMX/CLV | Yes |
| 20928 | 201608 | NA | NA | INH RIF EMB PZA CS MFX CAP PAS PTO | SM INH RIF RFP AM MFX AMX/CLV LVFX PA PAS RFB | No |
| 21097 | 201608 | NA | NA | INH RIF RFB EMB PZA MFX CAP PAS PTO | SM INH RIF RFO LVFX RFB | No |
| 21209 | 201609 | NA | NA | INH RIF EMB PZA PTO AM | INH RIF EMB SM PAS | No |
| 21367 | 201610 | NA | NA | INH RIF EMB PZA CAP MFX CS PTO LZD | AM INH RIF RFB CAP RFP SM | Yes |
| 21431 | 201610 | NA | NA | INH RIF EMB PZA CAP MFX PTO CLR | SM RIF RFP INH PA PAS RFB | No |

INH: isoniazid, RIF: rifampicin, EMB: ethambutol, PZA: pyrazinamide, SM: streptomycin, PAS: para-aminosalicylate, CAP: capreomycin, PA: isoniazid aminosalicylate, MFX: moxifloxacin, PTO: protionamide, CFZ: clofazimine, CLR: clarithromycin, AMX/CLV: amoxicillin/clavulanate, LVFX: levofloxacin, OFX: ofloxacin, AM: amikacin, RFP: rifapentine, GFX: gatifloxacin, CS: cycloserine, RFB: rifabutin, LZD: linezolid.

NA: Not Available.

**Table S2. Details of 30 CFZ-resistant clinical isolates.**

| ID | Date of isolation | Medication administration record | Drug resistance profile | CFZ treatment |
| --- | --- | --- | --- | --- |
| 11819 | 201303 | INH RIF RFP EMB PZA AM LVFX MFX RFB PAS PTO CLR | INH SM EMB RIF RFP LVFX OFX CFZ | No |
| 12458 | 201306 | INH RIF EMB PZA | INH SM EMB RIF RFB OFX LVFX RFP CFZ | No |
| 12881 | 201308 | INH RIF RFP EMB PZA AM LVFX MFX RFB PAS PTO CLR | INH SM EMB RIF RFP LVFX OFX CFZ | No |
| 14522 | 201404 | PA RFB EMB PZA GFX PTO CLR AMX/CLV MFX PAS CS CAP | OFX LVFX AM RIF INH EMB RFB CAP RFP SM CFZ | Yes |
| 14916 | 201407 | INH RIF EMB PZA SM AM LVFX MFX CAP PTO | OFX LVFX RFP AM RIF INH EMB RFB CAP RFP SM CFZ | Yes |
| 17402 | 201507 | INH RIF | LVFX MFX RIF INH EMB RFB CAP RFP SM PTO CFZ | No |
| 19405 | 201602 | INH RFB EMB PZA CS AK LVFX MFX CPM PAS PTO CLR | INH SM EMB RIF AM OFX LVFX PAS RFP RFB CFZ | Yes |
| 19477 | 201602 | INH RIF EMB PZA | INH SM RIF RFP RFB CFZ | No |
| 19524 | 201603 | INH RIF EMB | CFZ | No |
| 19526 | 201603 | INH RIF EMB PZA CS MFX | OFX LVFX RIF INH EMB RFB CAP RFP SM CFZ | No |
| 19531 | 201603 | / | AMX/CLV CFZ | No |
| 19552 | 201603 | INH RIF EMB PZA MFX CAP PAS PTO | LVFX AM MFX AMX/CLV RIF INH EMB RFB RFP CAP PAS SM OFX CFZ | No |
| 19562 | 201603 | INH RFP LVFX | LVFX CLR MFX AMX/CLV INH CFZ | No |
| 19575 | 201603 | INH RIF EMB | CFZ | No |
| 19579 | 201603 | / | MFX AMX/CLV CFZ | No |
| 19623 | 201603 | INH RIF EMB PZA RFP LVFX | MFX AMX/CLV SM CFZ | No |
| 19624 | 201603 | / | MFX AMX/CLV CFZ | No |
| 19629 | 201603 | / | MFX AMX/CLV CFZ | No |
| 19655 | 201603 | INH RIF EMB PZA | SM RIF RFP AMX/CLV INH RFB CFZ | No |
| 19681 | 201603 | CLR | MFX AMX/CLV LVFX CFZ | No |
| 19682 | 201603 | INH RIF PZA EMB | MFX AMX/CLV CFZ | No |
| 19683 | 201603 | / | MFX AMX/CLV CFZ | No |
| 19762 | 201603 | INH RIF EMB PZA AM LVFX MFX CAP PAS PTO | SM RFP AM PTO MFX AMX/CLV INH LVFX CAP PA RFB CFZ | No |
| 19821 | 201604 | INH RIF RFB EMB PZA PA MFX RFB | SM RIF RFP AM PTO MFX AMX/CLV INH EMB CAP PA RFB CFZ | No |
| 19872 | 201604 | INH RIF SM EMB PZA PTO | RIF RFP PTO MFX AMX/CLV INH LVFX CAP PA RFB CFZ | No |
| 19905 | 201604 | / | AMX/CLV CFZ | No |
| 19960 | 201604 | INH RIF PZA EMB PA LVFX PTO | SM RIF RFP AMX/CLV RFB CFZ | No |
| 19994 | 201604 | RFB AMX/CLV MFX PA PTO | RIF RFP AM PTO MFX AMX/CLV INH EMB LVFX PA RFB CFZ | Yes |
| 20053 | 201605 | INH EMB RIF PZA SM LVFX MFX RFB PTO PA | LVFX MFX AMX/CLV RIF INH RFB CAP RFP SM CFZ | No |
| 20155 | 201605 | / | AMX/CLV CFZ | No |

INH: isoniazid, RIF: rifampicin, EMB: ethambutol, PZA: pyrazinamide, SM: streptomycin, PAS: para-aminosalicylate, CAP: capreomycin, PA: isoniazid aminosalicylate, MFX: moxifloxacin, PTO: protionamide, CFZ: clofazimine, CLR: clarithromycin, AMX/CLV: amoxicillin/clavulanate, LVFX: levofloxacin, OFX: ofloxacin, AM: amikacin, RFP: rifapentine, CS: cycloserine, RFB: rifabutin, LZD: linezolid, GFX: gatifloxacin.
